# Professional appraisal of online information about children’s footwear measurement and fit: readability, usability and quality

**DOI:** 10.1186/s13047-020-0370-x

**Published:** 2020-01-14

**Authors:** Carina Price, Michael Haley, Anita Williams, Chris Nester, Stewart C. Morrison

**Affiliations:** 1 School of Health & Society, Brian Blatchford Building, Frederick Road Campus, University of Salford, Salford, M6 6PU UK; 2 University of Brighton, Eastbourne, BN20 7UR UK

**Keywords:** Shoes, Measurement, Paediatric, Internet, Advice

## Abstract

**Background:**

Parents increasingly use the internet to seek health information, share information and for purchasing textiles and footwear. This shift in footwear purchasing habits raises concern about how (and if) parents are getting their children’s feet measured, and what support strategies are in place to support the fit of footwear. In response to this, some companies and healthcare organisations have developed resources to support home measurement of foot size, and link these measures to footwear selection, measurement and fitting. The aim of this research was to undertake an appraisal of web-based resources about measurement and fit of children’s footwear, focussing specifically on readability, usability and quality.

**Methods:**

Search terms relating to children’s foot measurement were compiled and online searching was undertaken. Search results were saved and screened for relevance. Existing resources were categorised based on their source e.g. a footwear company or a health website. The 15 most commonly identified resources were reviewed by a professional panel for readability, content, usability and validity. One researcher also assessed the accessibility and reading ease of the resources.

**Results:**

Online resources were predominantly from commercial footwear companies (54%). Health information sources from professional bodies made up 4.2% of the resources identified. The top 15 resources had appropriate reading ease scores for parents (SMOG Index 4.3–8.2). Accessibility scores (the product of the number of times it appeared in search results and its ranking in the results) were highest for commercial footwear companies. The panel scores for readability ranged from 2.7 to 9 out of 10, with a similar range for content, usability and validity.

**Conclusions:**

Information for parents seeking to purchase footwear for their children is readily available online but this was largely dominated by commercial footwear companies. The quality and usability of this information is of a moderate standard; notable improvements could be made to the validity of the task the child is asked to undertake and the measures being taken. Improvements in these resources would improve the data input to the selection of footwear and therefore have a beneficial impact on footwear fit in children.

## Background

Parents are known to seek health information online [1] and increased use of technology has supported information sharing through websites, forums and social media [2, 3]. In the United Kingdom in 2018, 54% of parents described using the internet to look for health-related information [4]. In relation to footwear purchases, the internet has supported a considerable shift from in-store to online purchasing which accounted for over 19% of total sales in the British footwear, clothing, and textile industry in 2018 [5]. Recent work exploring parents’ knowledge, practices and perceptions of children’s feet identified that parents wanted accurate, clear and consistent foot health information [6]. This work also highlighted the challenges with footwear choices in early childhood and identified the influence of footwear retailers in promoting information about foot development and footwear choices.

Online purchasing of footwear is increasingly common and may pose challenges with ensuring that children have their feet measured prior to purchase as it negates the opportunity to try the footwear prior to purchase. To offset the expense to the companies associated with return of unsuitable purchases, many offer online fitting tools and advice such as size guides and printable charts to aide purchasing choices. For parents to make informed footwear choices this information needs to be accessible and, from a professional perspective, credible as this could have implications for promoting foot health in children [7]. Previous research advises that parents are unsure of how to evaluate the reliability of online health resources for their children [1] and that less than 10% of parents ‘*greatly trust*’ health information that they have identified through internet search engines [2]. These concerns are supported with the results of a systematic review which identified the quality of online health information as low [8];, a similar outcome to a review of scientific information on the internet [9]. Considering footwear information specifically, parents desire to purchase footwear online [4], access to online health information [4] and the prevalence of such information [6] highlight that online resources can influence parents. The quality and accessibility of this type of information is key for parents to be able to find, digest, understand and implement the tools to assist their purchasing behaviours; ultimately affecting the fit and therefore the appropriateness of the footwear their children will wear, which has wider implications for promoting foot health in children [7]. Inappropriate footwear choices in childhood could impact on foot development and health [10–13], although the mechanisms for these effects are not clear. Immediate effects of ill-fitting footwear are evident in the gait of infants and children in that shoes that are too large have been shown to affect spatial and temporal gait parameters [14] leading to greater instability during walking. Furthermore, shoes that are too big have been reported to impact on hip, knee and ankle kinematics during walking [15] and parents commonly believe footwear to be causal in the development of foot complaints [7].

Unlike footwear designed for adults, the footwear design for children needs to consider appropriate dimensions for growth. Foot length will increase by 2 mm per month up to three years of age and from five to 12 this decreases to 0.8–1 cm per year [13, 16]. This requires adjustments to the last and the fit process of children’s footwear. It also means that some sources recommend children having their feet measured every 6 weeks to 6 months, dependent on their age. Despite recommendations, a survey of children’s foot size in a shoe store identified that 12.5% of the children were wearing shoes that were at least a size too small [17]. In more widespread surveys the number of children reportedly wearing poorly sized shoes is more than half [10, 18] and this is even higher in children with disability [19]. With this high prevalence of incorrectly fitting footwear in children and hence associated foot problems, providing reliable and high-quality advice to parents through accessible sources to enable them to make informed footwear purchasing. The aim of this research was to undertake an appraisal of web-based resources about measurement and fit of children’s footwear (up to 12 years of age), focussing specifically on readability, usability and quality.

## Methods

**Fig. 1:**
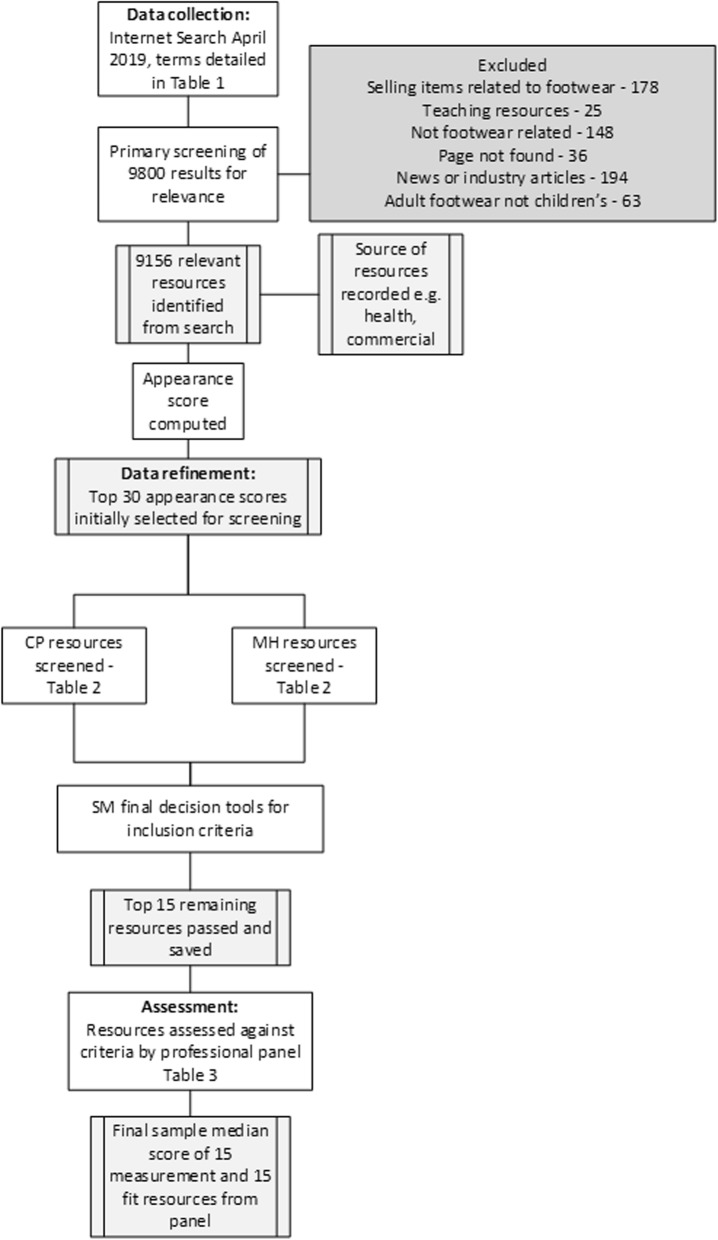
Flow chart for paper methodology: resource search, screening and assessment of resources by professional panel Note: authors are identified by initials at each key stage.

The methods adopted in this study are presented in Fig. 1.

### Data collection

Search terms relating to children’s foot measurement and fitting information were obtained from our existing work where terms were reviewed and agreed with a panel of  parents and clinicians [6]. For this study, search terms were compiled as a child or stage term plus a footwear term and a fit term (Table 1). The original keywords were expanded to include more nouns relating to children of different ages and developmental stage (e.g. Toddler, Infant), similarly footwear terms for specific milestones were added (e.g. “First shoes”, “School shoes”). Terms relating to fit were expanded to provide wider scope relating to footwear. These were agreed by the research team (Table 1).

**Table 1:** Search terms for online resources searches

Child term	Foot or Footwear term	Fit term
Child*	Shoe (s)	Size
Kid (s, ‘s)	Footwear	Sizing
Infant (s, ‘s)	Foot	Size chart
Bab*		Fit
Toddler (s, ‘s)		Fitting
Stage term		Information
First		Advice
School		Help
Pre-walker		Resource
Cruising		Guide
		Tools
		Measure
		Scale

The * signifies a truncation: baby, babies

Searching was undertaken using Google search engine by one researcher (CP) in a single week (beginning 29th April 2019). Cookies were turned off on the web browser prior to searches and the cache was cleared at the beginning of searching and then after each cycle of child terms (after every 15 searches). The top 10 search results for each search term were output and saved using a search capture plug-in (Session Buddy, SessionBudy.com, Colorado, USA). Search results returned as adverts were ignored. These searches resulted in a primary set of 9800 resources which were screened for relevance (e.g. webpages were not related to footwear or function) (Fig. 1).

Following this, 9156 resources remained, and these were categorised based on the source of the material (for example from a commercial footwear company, a health website or general such as a newspaper). After categorisation an appearance score was computed by rating the resources (10–1) for their rank following the search and summing this number for the total number of appearances. For example, a resource which appeared in three searches and as the second result in two of these and ninth in one would be scored 20 (the sum of 9, for being second rank, 9, for being second rank and 2, for being ninth rank). This resulted in scores which were a function of both the number of times the resource appeared using the key words and how high the resource appeared in the google search.

The top 30 resources for appearance score were initially selected for screening and recorded by the researcher as PDF documents representing each resource alongside the links for the associated webpages.

### Data refinement

These 30 resources were screened for inclusion and exclusion criteria (Table 2) by two researchers (CP and MH).

**Table 2:** Inclusion and exclusion criteria for resources and criteria for categorisation as foot measurement

Inclusion Criteria	Exclusion Criteria
Text in English	Text written in non-English language
UK based or directed source	Not UK based and relating specifically to footwear sizes which are not UK standard, e.g. EU or US sizing
Content relating to children aged up to 12 years of age	Content relating to adults or clinical groups
Directed to parents/carers of children	Directed to the footwear industry or clinicians
Advice relating to foot measurement	Only describes shoe sizes etc. and does not expand providing advice or description
Open to access by members of the public with no registration or subscription	Required a subscription to be able to access all primary and secondary information

Disagreement in terms of inclusion criteria or resource source was to be decided a third member of the research team (SM), however this was not required. This resulted in 15 resources which had passed inclusion criteria.

### Assessment

The professional panel was composed of 4 professionals working within footwear related roles (SM – PhD paediatric podiatry, CN – PhD biomechanics, AW – PhD footwear, MH – PhD candidate footwear) and all currently working on topic-related research projects. These roles included experience within clinical practice, research, footwear industry and academia. All four offered a breath of knowledge of the topic and were considered to be in a suitable position to comment on the resources, which previous literature has told us parents do not feel that they are in a position to do [1]. The professional panel rated the resources within two months of the original searches being completed.

The professional panel received the resources in a web format such that the full usability of the resource was available as well as a PDF backup in case the website had been withdrawn. They also received criteria for assessment with the scoring guide (Appendix) and associated instructions to help them rate the resources. The criteria for assessment were defined by the research team but predominantly CP who was not on the professional panel. This was designed through scoping literature assessing health related websites, in particular previous research which assessed the quality of websites that parents accessed for advice about their child’s development [20] (Table 3). At the same time as the professional panel undertook their review of the resources, aspects relating to readability were quantified and recorded by CP (not a member of the professional panel) using the SMOG Index calculated with an online tool (www.readabilityformulas.com/smog-readability-formula.php). The SMOG index is a readability score [21] which estimates the years of education required to be able to understand a piece of written text. This scoring reflects US grade levels within school and therefore provides an approximate age in years of a reader who can fully understand the text [21]. This system is the preferred method approach to determining the readability of healthcare material [22] and has broad application across healthcare research [23–25]. To assist in interpretation of this paper, the age relating to the US grades will be referred to as school grading systems are not consistent.

**Table 3:** Criteria for assessment alongside description of assessment

Criteria for assessment	Score	Description
Accessibility	Total score for searches and no. of times as first in search	How many search terms returned this resource and how easy is it to find?
Reading ease	SMOG Index	Readability scores calculated with the SMOG Index.
Readability	Rated out of 10	Are the instructions clear?
Content	Rated out of 10	Advice and/or quantitative measure? Clear layout and presence of diagrams or images.
Usability	Rated out of 10	How it loads online and opens – can you use it on a tablet or phone? Does it need printing? How easy is the website to navigate? Are the buttons clear and is the layout easy to read?
Validity - task	Rated out of 10	Is the task or process described appropriate for measuring the feet of children?
Validity - measures	Rated out of 10	Which aspects of the foot does it quantify? E.g. length only, width measures, instep, whole foot with an app.

Once all scores were received, these were combined for all professional panel members for each resource and aspect. These were used to compute a median and inter-quartile range to describe the score for each aspect and resource from the panel. Resources are presented as resource 1–15 based on their overall accessibility score however, resources were not provided to the professional panel in this manner to prevent any bias associated with this value. Additional data outcomes included the source of the resources and appearance scores for each resource.

## Results

**Fig. 2:**
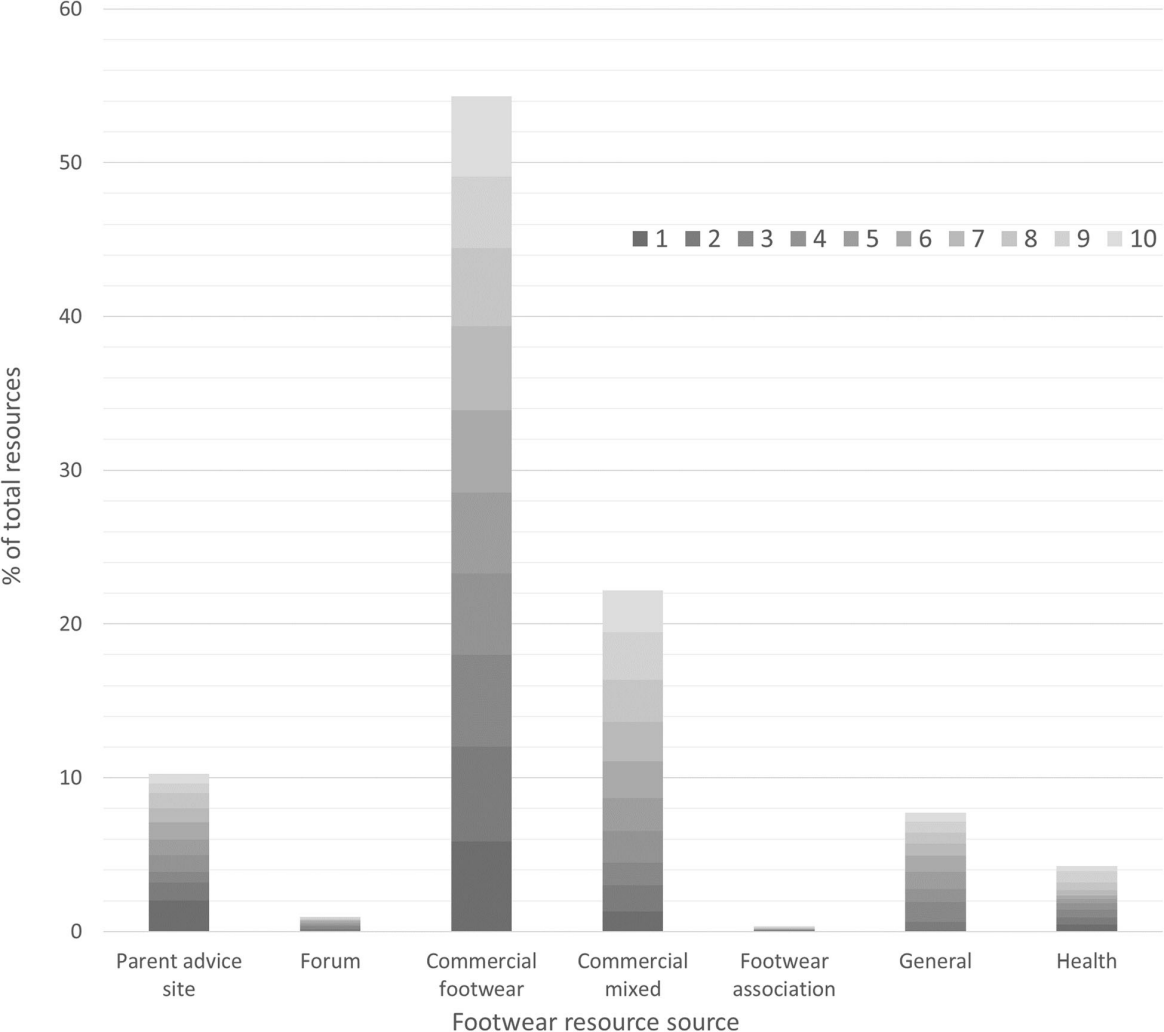
Screened resources categorised into footwear resource source with position returned in search engine identified with the grayscale

The source of the screened foot measurement and footwear fitting resources was identified (Fig. 2) and resources were predominantly from commercial footwear companies (54%). A large percentage of these commercial footwear resources were returned in the top three searches from the search engine; 18% of the total resources identified. Commercial websites from mixed stores such as department stores made up 22% of the resources, followed by parent advice websites (10%). Health information sources from professional bodies made up 4.2% of the overall resources. Forums (1%) resulted in a lower number of resources than parent advice sites (10%) and footwear association sites accounted for only 0.3% of the resources identified.

The data from the professional panel review of the 15 most identified resources can be seen in Table 4. Ten of the 15 resources were commercial sites for footwear companies with a further three resources being mixed commercial sites, including clothing retailers and department stores. Accessibility scores ranged from 63 to 3990 (if a website would have been top of every search the maximum score would be 98,000) which demonstrated the frequency at which some of the more common resources appeared in searches as high. Within these accessibility scores the most commonly found resource to appear top of the search in google appeared 133 times, with two resources never appearing first in the searches undertaken.

**Table 4:** Outcomes of professional assessment for the 15 measurement resources

Resource	Footwear resource source	Criteria for assessment						
		Accessibility	Reading Ease (SMOG Index)	Readability	Content	Usability	Validity – Task	Validity - Measures
1	CF	3990 [133]	6.25	8.0 (0.8)	8.5 (1.0)	9.0 (2.3)	8.0 (0.8)	7.5 (1.6)
2	CF	1196 [17]	6.73	6.0 (0.5)	5.5 (1.3)	6.4 (1.0)	4.5 (1.3)	4.0 (0.5)
3	CM	1015 [8]	8.20	9.0 (2.3)	9.0 (0.5)	8.5 (1.3)	8.5 (1.5)	8.5 (1.5)
4	GE	1448 [32]	7.40	3.2 (1.6)	2.5 (1.3)	3.5 (1.5)	3.2 (1.2)	3.0 (0.8)
5	PA	1152 [52]	5.80	5.0 (6.0)	3.0 (1.1)	4.2 (2.1)	4.0 (1.5)	4.5 (2.0)
6	CF	657 [6]	7.70	6.5 (3.8)	4.9 (3.8)	5.0 (0.9)	4.5 (2.0)	4.5 (2.3)
7	CF	454 [2]	6.75	7.4 (2.5)	3.7 (2.0)	3.7 (2.0)	2.0 (2.1)	1.5 (1.3)
8	CF	619 [10]	4.30	2.7 (1.8)	1.5 (1.3)	2.0 (2.0)	1.0 (0.8)	2.0 (2.3)
9	CF	443 [1]	5.70	5.4 (3.3)	3.2 (0.5)	4.0 (0.3)	2.7 (1.8)	3.0 (2.3)
10	CM	355 [0]	6.60	7.5 (2.0)	6.5 (1.9)	6.0 (2.3)	3.5 (3.4)	3.5 (3.3)
11	CF	464 [4]	6.50	4.5 (3.3)	2.8 (1.1)	3.0 (1.2)	2.5 (1.4)	2.0 (2.1)
12	CM	63 [0]	5.00	7.5 (1.8)	7.5 (2.0)	6.0 (0.8)	7.0 (2.3)	6.5 (1.5)
13	CF	286 [6]	5.80	5.4 (3.1)	3.2 (1.2)	5.5 (1.4)	3.0 (2.3)	3.0 (2.3)
14	CF	313 [13]	6.30	5.0 (4.3)	4.5 (2.5)	3.5 (3.8)	3.5 (3.3)	3.0 (2.5)
15	CF	102 [1]	6.70	2.7 (2.6)	3.0 (0.8)	3.0 (0.5)	2.0 (0.8)	2.5 (1.3)

Data presented as median (inter-quartile range) of scores out of 10 apart from accessibility which is a total score of the searches undertaken [no. of times as first in searches] and reading ease which is a single SMOG Index value – relating to US school grades- calculated by CP. Where footwear resources are categorised: *CF* commercial footwear, *CM* commercial mixed, *GE* general and *PA* parent advice

The reading ease scores computed using the SMOG index ranged from 4.3 to 8.2. This represents interpretation from age 8–9 years and ‘*easy to read*’ to age 13–14 years and ‘*fairly difficult to read*’. Three of the resources required a reading age of 12 years or above (see Table 4 - resources 3,4 and 6). The professional panel rated the readability, usability and quality (validity and content) of the resources with quite wide ranges for the assessment criteria across all of those identified. For validity, the average task and measures scores tended to be relatively consistent for each resource, those scoring higher in one (e.g. resource 1) scored higher in the other and those which scored lower in task validity (e.g. resource 8) reflected this in measure validity too. Across all data, the lowest median score was 1 and the highest was 9. For readability, the median resources scores ranged from 2.7 to 9 out of 10 with a similar range in the other criteria for assessment. Notable resources were 8, 9 and 15 which scored particularly low, resource 8 did not have a median score above 2.7 for any of the assessment criteria. In contrast, resources 1 and 3 scored highly across all criteria with the lowest scores being 7.5 and 8.5 respectively, both for validity measures. However, resource 3 had a reading ease score of 8.2. Despite scoring high for validity, the content would only be accessible by an audience with an older reading age and may mean that this resource was less easy to access.

## Discussion

There was a breadth of information to support foot measurement for footwear fitting online (and published) from various sources. The high number of search results for footwear companies (76%) compared to health care providers (4%) reflected the dominance of information presented to parents. This is consistent with previous literature exploring parents’ knowledge, practices and health-related perceptions of children’s feet [6]. This work described parents’ behaviour as the outcome of long-standing familiarity with brands, including their own experiences as children. Contrary to this, the low return of healthcare sources is concerning as these resources are those which parents perceive to be providing accurate and reliable information [26], although many parents were unsure about how to assess this [1]. Web users typically access resources at the top of their search results [27], and this usage varies depending on the types of device(s) used for the search. Despite accounting for only 4.2% of the results returned from the searches, over a third of healthcare resources ranked within the top three of the results returned in each search. This would suggest that health resources were visible, but we acknowledge that access to these would depend on the terminology entered into the search. Search engine optimisation might be an important consideration for healthcare providers and footwear association(s) to enhance the visibility of impartial and credible resources.

The dominance of commercial sources in the search results confirm that the professional panel had a focus on commercial footwear fit for the sites they reviewed; 10/15 were commercial footwear, 3/15 commercial mixed. No health, footwear association or forum sites appeared frequently enough that they were included for the final screening. The dominance of commercial sources was also reflected in high accessibility scores. The most returned resource was from a commercial footwear company and had a score of 3990, being the product of the number of times it was identified in the search and the position in which it was returned in the search results. For this resource 133 of these appearances were as the first item in the search, which was the highest by at least threefold. The lowest accessibility score was 63 with zero first position appearances, which was a commercial mixed resource. This demonstrated a difference in terms of how commonly a parent would identify each resource while searching. Again, this demonstrates that the foot measurement information is dominated by a few commercial footwear companies.

The accessibility and interpretation of published information is important for parents to comprehend the information they require to inform their footwear habits. The reading ease scores were appropriate for most of the resources in terms of interpretation and understanding. The highest score was equivalent to a reading age of 13–14 years of age, which suggests a higher complexity of the text and extends beyond typical recommendations [24]. It is important that written (health) education materials are accessible, at the lowest reading level that conveys true and accurate the information [28]. The majority of the resources align well to recommendations that resources with a reading age of more than 12 years should be rewritten to broaden the audience [29, 30]. In this study, readability was also assessed by the professional panel with a highest score of 9.0 (2.3) for the 3rd most identified resource (a mixed commercial source). This means all steps were clearly and concisely worded and followed a sequential pattern. The lowest score was 2.7 for both resource 15 and 8, both from commercial sources which means that, despite reading ease being appropriate for parents, the wording may be confusing and unclear, and instructions did not flow /make sense. This could result in confusing messages and inconsistencies for the parents, which may result in a distrust the resources or difficulty in its interpretation. This could potentially lead to problems with inaccurate foot measurements or poor footwear fitting, which could have longer term implications. Foot measurement is a skill and instructions for an untrained parent to be able to undertake such measures accurately enough to select a shoe size must be precise and clear.

Resource content including the use of quantitative measures, diagrams or images and a clear layout was the lowest rated aspect across all resources (median 3.7/10) Scores are impacted by website design and use of text, imagery and instructional videos. These relate to usability scores associated with use across multiple platforms and the need to print resources. The latter allow a child to stand and have their foot length/width marked and measured. Mobile applications were not included within the current search terms which may have identified further approaches such as generating a 3D image of the foot from photogrammetry [30]. Whilst the accuracy of these limited measures may be adequate for sizing, whether these measures alone (e.g. just heel to toe length and forefoot width) can enable correct footwear size and style selection is unclear [31].

The professional panel was used because parents report being unsure about how to assess the reliability of online health resources [1, 6]. Academics, clinicians and a footwear company employee were involved as they were experienced enough to address the validity of the task and measurement being undertaken and determine whether it was appropriate for measuring footwear fit (Table 3). These data encompassed a large range of values; however, the two aspects of the validity being assessed (task and measures) tended to score relatively consistently across each resource. This identified that resources that had a suitable task for assessment (e.g. standing still and weight bearing) then undertook appropriate measures while the child was in this position (e.g. measure of multiple foot aspects such as length, width and girth). Resources which quantified only unidimensional features of the foot such as length were scored lower, as were resources which measured the foot in a non-weightbearing position. In addition to this, the translation of these values to a shoe size is integral to the child receiving footwear of the correct size. The interpretation of foot measures and conversion to a shoe size occurs within the footwear company based on the measurements provided by the parent. This process requires further investigation to understand the association with fit. The consensus within the industry would be that for appropriate footwear fit, feet should be measured by an experienced shoe fitter who has been appropriately trained. The transition from in-store purchasing to online purchasing will mean that parents will move towards online fitting solutions as opposed to visiting store staff. Identifying a consensus approach for the footwear industry to employ to improve accuracy and reduce errors that result in ill-fitting footwear would reduce confusion for parents, as each website would suggest the same task and the same measures to fit footwear.

Some limitations to this research include using Google as the sole search engine, however more than 87% of UK users chose this as their primary search engine therefore this covers a significant number of searches that are undertaken in the UK [32]. These resources are aimed at parents yet have been reviewed for usability by academic researchers and clinicians. These professional panel members were in a position to comment on the validity however, further work exploring how parents rank the usability, accessibility and credibility of the information would help to progress the findings from this study. Also, a measure of which resources are being used and implemented by parents would help the translation of the findings from the current research to improve the tools which parents are utilising.

## Conclusions

Parents are increasingly using the internet to search for information about their children’s feet and to purchase footwear. Information is available to parents seeking to purchase footwear, but this is largely dominated by resources from commercial footwear companies. The quality and usability of this information is of moderate standard, often of low quality, and whilst readability was appropriate, content was inconsistent in terms of value in assisting footwear fit. Improvements are needed to help parents make informed decisions.

## Data Availability

The datasets generated and/or analysed during the current study are not publicly available but are available from the corresponding author on reasonable request.

## Appendix

**Table 5:** Criteria for assessment with the scoring guide

Criteria for assessment		Score 0 to 3.3	Score 3.4 to 6.7	Score 6.8 to 10
Accessibility	How many search terms returned this resource aka how easy is it to find?	Scored by CP	Scored by CP	Scored by CP
Reading Ease	Readability scores calculated with the SMOG Index.	Scored by CP	Scored by CP	Scored by CP
Readability	Are the instructions clear?	Wording is confusing and unclear. Instructions do not flow and might not make sense.	Instructions are relatively clear, but some points do not make sense or are not sequential.	All instructions or steps are clearly and concisely worded and follow a clear, sequential pattern
Content	Advice and/or quantitative measure?	Only subjective descriptions.	Mixed subjective and objective descriptions	Numerous objective tests as well as subjective descriptions to aid interpretation
	Clear layout and presence of diagrams or images.	Long paragraphs of text with no illustrations or clear points.	Clear points or tables in text.	Animation, diagrams and figures which help convey messages clearly.
		No, or few, images	Some images for description.	All, or most, points are followed with an image for clarity.
Usability	How it loads online and opens – can you use it on a table or phone?	Guide is not able to be used on phone/tablet or all devices attempted.	Guide worked on most platforms with most functionality	Guide worked effectively and consistency on all platforms attempted
	Does it need printing?	Guide needs printing and there is no scale to check print quality	Guide needs printing and there is a scale to check print quality.	Guide is fully functioning as an online tool and does not require any printing.
	How easy is the website to navigate? Are the buttons clear and is the layout easy to read?	Website is unclear to navigate. Multiple mouse clicks are required to access all information.	Website is relatively clear to navigate and some secondary pages are easy to find. Some secondary information is readily available, but not all.	Website is very clear and easy to navigate and secondary pages are found easily. Information and content of resources is accessible with 1 mouse click.
Validity - task	Is the task or process described appropriate for measuring the feet of children?	The task or process described is not what I consider essential for foot measurement in children.	Some of the task or process described is what I consider essential for foot measurement in children, but not all.	The task or process described is all of what I consider essential for foot measurement in children.
Validity - measures	Which aspects of the foot does it quantify? E.g. length only, width measures, instep, whole foot with an app.	Few of the key measures I associate as essential for foot measurement are included.	Most of the measures I associate as essential for foot measurement are included.	All key measures I associate as essential with foot measurement are included.
